# Expansion of KPC-producing Enterobacterales in four large hospitals in Hanoi, Vietnam

**DOI:** 10.1016/j.jgar.2021.09.007

**Published:** 2021-12

**Authors:** Tran Dieu Linh, Nguyen Hoai Thu, Keigo Shibayama, Masato Suzuki, LayMint Yoshida, Pham Duy Thai, Dang Duc Anh, Tran Nhu Duong, Hong Son Trinh, Vu Phuong Thom, Luu Thi Vu Nga, Nguyen Thi Kim Phuong, Bui Thanh Thuyet, Timothy R. Walsh, Le Viet Thanh, Anne-Laure Bañuls, H. Rogier van Doorn, Tran Van Anh, Tran Huy Hoang

**Affiliations:** a National Institute of Hygiene and Epidemiology, Hanoi, Vietnam; b National Institute of Infectious Diseases, Tokyo, Japan; c Institute of Tropical Diseases, Nagasaki University, Nagasaki, Japan; d Viet Duc Hospital, Hanoi, Vietnam; e Saint Paul Hospital, Hanoi, Vietnam; f Thanh Nhan Hospital, Hanoi, Vietnam; g 108 Military Central Hospital, Hanoi, Vietnam; h Department of Medical Microbiology and Infectious Disease, Cardiff University, Cardiff, UK; i Oxford University Clinical Research Unit, Hanoi, Vietnam; j Quadram Institute Bioscience, Norwich Research Park, Norwich, Norfolk, UK; k MIVEGEC (IRD-CNRS-Université de Montpellier), Centre IRD, Montpellier, France; l Centre for Tropical Medicine and Global Health, Nuffield Department of Clinical Medicine, University of Oxford, Oxford, UK; m Hanoi Medical University*,* Hanoi*,* Vietnam

**Keywords:** Carbapenem resistance, KPC, Enterobacterales, Vietnam

## Abstract

**Objectives:**

: The incidence of carbapenem resistance among nosocomial Gram-negative bacteria in Vietnam is high and increasing, including among Enterobacterales. In this study, we assessed the presence of one of the main carbapenemase genes, *bla*_KPC_, among carbapenem-resistant Enterobacterales (CRE) from four large hospitals in Hanoi, Vietnam, between 2010 and 2015, and described their key molecular characteristics.

**Methods:**

: KPC-producing Enterobacterales were detected using conventional PCR and were further analysed using *S1* nuclease pulsed-field gel electrophoresis (S1-PFGE), Southern blotting and whole-genome sequencing (WGS) for sequence typing and genetic characterisation.

**Results:**

: *bla*_KPC_ genes were detected in 122 (20.4%) of 599 CRE isolates. *bla*_KPC_-carrying plasmids were diverse in size. *Klebsiella pneumoniae* harbouring *bla*_KPC_ genes belonged to ST15 and ST11, whereas KPC-producing *Escherichia coli* showed more diverse sequence types including ST3580, ST448, ST709 and ST405. Genotypic relationships supported the hypothesis of circulation of a population of ‘resident’ resistant bacteria in one hospital through the years and of transmission among these hospitals via patient transfer. WGS results revealed co-carriage of several other antimicrobial resistance genes and three different genetic contexts of *bla*_KPC-2_. Among these, the combination of IS*Ecp1*–*bla*_CTX-M_ and IS*Kpn27*–*bla*_KPC_–ΔIS*Kpn6* on the same plasmid is reported for the first time.

**Conclusion:**

: We describe the dissemination of *bla*_KPC_-expressing Enterobacterales in four large hospitals in Hanoi, Vietnam, since 2010, which may have started earlier, along with their resistance patterns, sequence types, genotypic relationship, plasmid sizes and genetic context, thereby contributing to the overall picture of the antimicrobial resistance situation in Enterobacterales in Vietnam.

## Highlights

• 122 (20.4%) of 599 carbapenem-resistant Enterobacterales (CRE) isolates carried *bla*_KPC_ genes*.*
• Predominance of ST15 *K. pneumoniae*, whereas *E. coli* presented more diverse sequence types.
• *bla*_KPC_-bearing plasmids were diverse in size.
• Three different models of genetic context of *bla*_KPC-2_.
• Hypothesis of circulation of resistant bacteria and transmission among hospitals.

## 1 Introduction

The incidence of carbapenem resistance among nosocomial pathogens in Vietnam is high and increasing, especially among *Escherichia coli* and Gram-negative ‘ESKAPE’ organisms (*Klebsiella pneumoniae, Acinetobacter baumannii, Pseudomonas aeruginosa* and *Enterobacter* spp.). Data from a nationwide hospital surveillance network in 2016–2017 showed that rates of carbapenem resistance among *K. pneumoniae, E. coli* and *Enterobacter* spp*.* were 29%, 11% and 27%, respectively [1]. A recent study from Vietnam including more than 2200 patients admitted to 12 hospitals throughout the country during 2017 and 2018 reported that 52% of patients were colonised with carbapenem-resistant Enterobacterales (CRE) [2]. Since the first NDM-1-producing *K. pneumoniae* in Vietnam was isolated from the urinary tract of a 62-year-old hospitalised patient in 2010 [3], most class A, B and D carbapenemases in Enterobacterales have been reported from Vietnam [4], [5], [6]. In the southern part of Vietnam, carbapenem-resistant *K. pneumoniae* clinical isolates producing various carbapenemases such as KPC-2, NDM-1, NDM-4 and OXA-48 have been described [6,7]. Studies from three hospitals in Hanoi, in the northern part of Vietnam, detected diverse variants of carbapenemase genes such as KPC-2, KPC-3, KPC-4, NDM-1, IMP-4, IMP-79, VIM-1 and OXA-48 in Enterobacterales [4,5,8,9]. Data on the molecular characteristics of carbapenemases from carbapenemase-producing Enterobacterales (CPE) in clinical isolates in Vietnam, a lower-middle income country with a high and increasing burden of antimicrobial resistance and hospital-acquired infections, are still sparse. Here we present the phenotypic and molecular characteristics of KPC-producing CPE isolates from four major hospitals in Hanoi between 2010 and 2015 in order to gain a better understanding of the circulation of CPE in Vietnam and to compare this with local, regional and global data to add to the current knowledge base*.* Our results will contribute to outline a larger picture of CPE in Vietnam and will serve as important scientific information for government action plans on antibiotic resistance control.

## 2 Materials and methods

### 2.1 Study sites and sample collection

We prospectively collected CRE isolates from four large hospitals including Saint Paul (A), Thanh Nhan (B), Viet Duc (C) and 108 Military Central Hospital (D) located in the centre of Hanoi, Vietnam. All four are public hospitals; three are general hospitals (A, B and D) and C is a specialised hospital for surgery. A and B are city hospitals with a 600-bed capacity, while C and D are central hospitals with a greater than 1000-bed capacity. Demographic and basic clinical information of patients whose specimens were positive for CRE were collected from clinical notes and included age, sex, date of admission, clinical diagnosis, origin of collected sample, date of sample collection and culture results. Treatment and clinical outcome data were not available for this study.

CRE isolated from clinical specimens were tested for antimicrobial susceptibility at the four sites by the disk diffusion method according to Clinical and Laboratory Standards Institute (CLSI) guidelines [10,11]. Microbiology laboratories in the four hospitals were requested to collect and send all bacterial isolates resistant to at least one carbapenem to the National Institute of Hygiene and Epidemiology for further characterisation (*n* = 599, including 179 isolates from hospital A, 87 from hospital B, 95 from hospital C and 238 from hospital D).

### 2.2 Antimicrobial susceptibility testing and detection of antimicrobial resistance genes

Minimum inhibitory concentrations (MICs) were determined centrally by the agar dilution method for imipenem, meropenem, cefotaxime, ceftazidime and ciprofloxacin and by microdilution for colistin (Sigma-Aldrich) according to CLSI and European Committee on Antimicrobial Susceptibility testing (EUCAST) guidelines [11,12]. *Escherichia coli* ATCC 25922 and *P. aeruginosa* ATCC 27853 were used as control strains.

Collected isolates were screened for four common carbapenemase genes, including *bla*_KPC_, *bla*_NDM-1_, *bla*_IMP_ and *bla*_OXA-48_, as well as three other common β-lactamase-encoding genes (*bla*_TEM_, *bla*_SHV_ and *bla*_CTX-M_) as described previously [4]. Resulting amplicons were sequenced using conventional Sanger sequencing.

### 2.3 Multilocus sequence typing (MLST)

MLST was done using PubMLST for all *bla*_KPC_-positive isolates (*n* = 122) [13]. Briefly, seven housekeeping genes were amplified by PCR, sequenced and compared with the sequences submitted to the MLST database to determine the sequence type (ST).

### 2.4 S1 nuclease pulsed-field gel electrophoresis (S1-PFGE) and Southern blotting

*bla*_KPC_-positive isolates were further analysed using S1-PFGE with *Xba*I-digested *Salmonella enterica* serovar Braenderup H9812 as a reference molecular weight marker on a CHEF-DR III platform (Bio-Rad, Hercules, CA, USA), followed by membrane transfer and hybridisation with labelled probes in an HL-2000 HybriLinker Hybridization Oven (UPV, Germany). Autoradiograms were visualised according to standard Southern blotting protocols [14].

### 2.5 Whole-genome sequencing (WGS) and characterisation of the genetic environment of bla_KPC_ genes

A subset of *bla*_KPC_-carrying CPE isolates (*n* = 69) was selected for further analysis including representatives of different hospitals, collection years, departments and sample types. Genomic DNA libraries of selected strains were prepared for WGS using a Nextera XT DNA Library Preparation Kit (Illumina Inc., San Diego, CA, USA) according to the manufacturer's instructions. Then, 300-bp paired-end sequencing was performed on an Illumina MiSeq platform (MiSeq Reagent Kit v3; 600 cycles). Raw sequence reads were de novo assembled into contigs using SPAdes v.3.9.0 with pre-defined Kmers set. Antimicrobial resistance genes were identified using ResFinder v.2.1, and MLST profiles were generated using the platform of the Center for Genomic Epidemiology, Technical University of Denmark, coupled with the PubMLST.org database.

Plasmids were identified and typed using PlasmidFinder v.1.3. Structures of genetic contexts surrounding *bla*_KPC_ genes were mapped using ISfinder, BLASTN v.2.6.0 and genoPlotR v.0.8.3.

Phylogenetic trees based on the core genome single nucleotide polymorphisms (SNPs) were constructed from WGS data of the 60 *bla*_KPC_-carrying *K. pneumoniae* and 9 *bla*_KPC_-carrying *E. coli* isolates using Parsnp 1.2 and IQ-TREE 2.0 [15,16].

### 2.6 Statistical methods

Isolates and patient data were analysed in Microsoft Excel 2017 (Microsoft Corp., Redmond, WA, USA) using descriptive statistics as appropriate.

## 3 Results

### 3.1 Distribution of bla_KPC_-positive carbapenemase-producing Enterobacterales (CPE) isolates among hospitals and their phenotypes

During the study period, 599 CRE were collected in four hospitals, comprising 64 isolates in 2010, 97 isolates in 2011, 64 isolates in 2012, 68 isolates in 2013, 149 isolates in 2014 and 157 isolates in 2015. Among these, *K. pneumoniae* (*n* = 305) and *E. coli* (*n* = 186) accounted for the majority of isolates; other bacteria included other *Klebsiella* spp. (*n* = 54), *Enterobacter* spp. (*n* = 29) and *Citrobacter* spp. (*n* = 25).

Of the 599 CRE isolates, 122 (20.4%) were positive for *bla*_KPC_, including 13 *E. coli* (10.7%) and 109 *K. pneumoniae* (89.3%). No *bla*_KPC_ gene was detected in the other bacteria. Hospital A contributed the largest number of *bla*_KPC_-positive isolates (*n* = 101, accounting for 82.8%), followed by hospital B (*n* = 9; 7.4%), hospital D (*n* = 8; 6.6%) and hospital C (*n* = 4; 3.3%) (Table 1). The first detected *bla*_KPC_-positive bacterium was *K. pneumoniae*, isolated from bronchial fluid of a ventilated patient in the intensive care unit (ICU) of hospital B on 16 January 2010.

**Table 1:** Distribution of *bla*_KPC_-positive Enterobacterales isolates among hospitals in Hanoi, Vietnam, from 2010–2015.

Hospital	No. of isolates						Total [no. (%)]
	2010	2011	2012	2013	2014	2015	
A	5	30	18	18	30	0	101 (82.8)
B	6	0	0	2	1	0	9 (7.4)
C	2	0	0	0	1	1	4 (3.3)
D	0	0	0	0	2	6	8 (6.6)
Total	13	30	18	20	34	7	122 (100)

From the clinical information collected, the clinical characteristics of *bla*_KPC_-positive isolates (*n* = 122) were revealed after de-duplication. The highest proportion was from the neonatal ICU (*n* = 97; 79.5%), followed by the ICU (*n* = 15; 12.3%), tuberculosis (TB) and lung diseases (*n* = 4; 3.3%) and four other departments (Table 2). The most dominant sample type among *bla*_KPC_-positive isolates was bronchial fluid (*n* = 92; 75.4%). Other types of sample included blood (*n* = 18; 14.8%), sputum (*n* = 6; 4.9%), urine (*n* = 4; 3.3%), pleural fluid (*n* = 1; 0.8%) and abdominal fluid (*n* = 1; 0.8%).

**Table 2:** Clinical and genotypic characteristics of *bla*_KPC_-positive Enterobacterales isolates (*n* = 122) among hospitals (A–D) in Hanoi, Vietnam, from 2010–2015.

Characteristic		No. of isolates				Total [no. (%)]
		A	B	C	D	
Department	Neonatal ICU	95	2	0	0	97 (79.5)
	ICU	6	7	2	0	15 (12.3)
	TB and lung diseases	0	0	0	4	4 (3.3)
	Urology	0	0	2	0	2 (1.6)
	Neurology	0	0	0	2	2 (1.6)
	Cardiology	0	0	0	1	1 (0.8)
	Thoracic surgery	0	0	0	1	1 (0.8)
Sample type	Bronchial fluid	86	5	1	0	92 (75.4)
	Blood	15	1	0	2	18 (14.8)
	Sputum	0	3	0	3	6 (4.9)
	Urine	0	0	2	2	4 (3.3)
	Pleural fluid	0	0	0	1	1 (0.8)
	Abdominal fluid	0	0	1	0	1 (0.8)
Carbapenemase-encoding gene	*bla*_NDM-1_	8	1	1	0	10 (8.2)
	*bla*_OXA-48_	1	0	0	0	1 (0.8)
	*bla*_IMP_	0	0	0	0	0 (0.0)
ESBL-encoding gene	*bla*_TEM_	99	7	4	6	116 (95.1)
	*bla*_SHV_	90	9	2	8	109 (89.3)
	*bla*_CTX-M_	33	8	2	2	45 (36.9)

ICU, intensive care unit; TB, tuberculosis; ESBL, extended-spectrum β-lactamase.

Based on sequencing results, 67 (97.1%) of a selected subset of 69 *bla*_KPC_-positive isolates harboured *bla*_KPC-2_, whereas the other 2 isolates harboured *bla*_KPC-12_ and *bla*_KPC-14_, both from hospital D. No *bla*_IMP_ gene was detected and one isolate harboured *bla*_OXA-48_. Ten isolates (six *K. pneumoniae* and four *E. coli*) co-carried *bla*_KPC_ and *bla*_NDM-1_ genes (Table 2). Almost all *bla*_KPC_-harbouring strains carried *bla*_TEM_ and *bla*_SHV_ genes [116 (95.1%) and 109 (89.3%), respectively], whilst *bla*_CTX-M_ genes were less common among these isolates [45 (36.9%)].

Results of MIC testing (Table 3) revealed that 110 (90.2%) and 98 (80.3%) isolates were resistant to third-generation cephalosporins (ceftazidime and cefotaxime, respectively) and 111 (91.0%) isolates were resistant to fluoroquinolones (ciprofloxacin). Moreover, 80 (65.6%) and 79 (64.8%) isolates were resistant to carbapenems (imipenem and meropenem, respectively) and 29 (23.8%) were resistant to colistin. All *E. coli* were susceptible to meropenem and colistin. No significant difference was detected in MICs among isolates carrying only *bla*_KPC_ genes and those co-carrying *bla*_KPC_ and other β-lactamase genes.

**Table 3:** Minimum inhibitory concentrations (MICs) of *bla*_KPC_-positive Enterobacterales.

Species	Hospital (no. of isolates)	Resistant phenotype MIC in μg/mL (no. of isolates)					
		IPM	MEM	CAZ	CTX	CIP	COL
*Klebsiella pneumoniae* (*n* = 109)	A (*n* = 90)	≥8 (63)	≥8 (69)	≥16 (83)	≥16 (75)	≥16 (83)	≥8 (24)
	B (*n* = 9)	≥16 (4)	≥16 (3)	≥64 (7)	≥16 (5)	≥8 (7)	≥4 (2)
	C (*n* = 2)	>64 (1)	>64 (1)	≥256 (1)	≥512 (1)	≥128 (1)	≥8 (1)
	D (*n* = 8)	≥4 (4)	≥4 (6)	≥64 (8)	≥128 (7)	≥64 (8)	≥16 (2)
*Escherichia coli* (*n* = 13)	A (*n* = 11)	>64 (6)	–	≥128 (9)	≥8 (8)	≥32 (10)	–
	C (*n* = 2)	≥4 (2)	–	>512 (2)	≥512 (2)	≥16 (2)	–
Total [no. (%) of isolates]	*n* = 122	80 (65.6)	79 (64.8)	110 (90.2)	98 (80.3)	111 (91.0)	29 (23.8)

MIC, minimum inhibitory concentration; IPM, imipenem; MEM, meropenem; CAZ, ceftazidime; CTX, cefotaxime; CIP, ciprofloxacin; COL, colistin.

### 3.2 Antimicrobial resistance gene profile, sequence typing and genotypic relationship

**Fig. 1:**
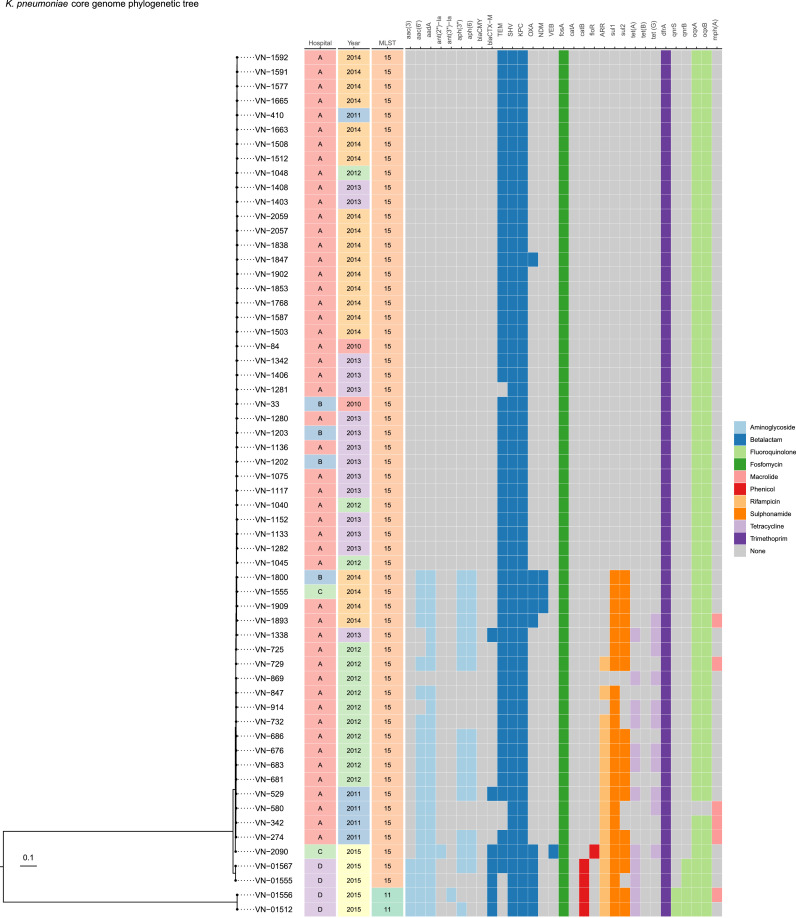
Core genome phylogenetic tree of *bla*_KPC_-carrying *Klebsiella pneumoniae* isolates. Presence of resistance genes was colour coded by different antibiotic categories, while grey blocks showed no corresponding resistance genes were detected.

**Fig. 2:**
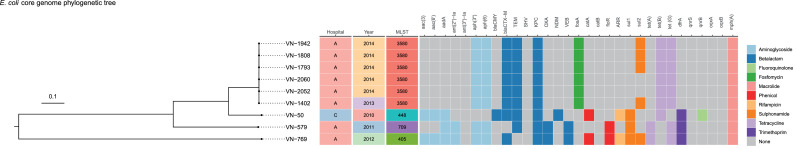
Core genome phylogenetic tree of *bla*_KPC_-carrying *Escherichia coli* isolates. Presence of resistance genes was colour coded by different antibiotic categories, while grey blocks showed no corresponding resistance genes were detected.

WGS data revealed the resistance profile of *bla*_KPC_-harbouring isolates to a wide range of antibiotics (Table 4). All isolates carried genes conferring resistance to at least three different antibiotic categories, but with considerable variation in the resistance-conferring elements carried. Indeed, two resistance genes (*fosA* and *dfrA*) were present in all *K. pneumoniae* isolates but not all *E. coli* isolates. On the other hand, the *mph(A)* gene was observed in all *E. coli* but only a few *K. pneumoniae* isolates (Fig. 1, Fig. 2). Notably, one *K. pneumoniae* isolate carried resistance genes for all 10 investigated antibiotic categories. All 60 (100%) *bla*_KPC_-carrying *K. pneumoniae* isolates carried resistance genes against β-lactams, fosfomycin and trimethoprim. Resistance elements to aminoglycosides and quinolones were also detected in high proportions (≥95%) among *K. pneumoniae* isolates; however, few strains had genotypic resistance to macrolides. In *E. coli*, all nine (100%) *bla*_KPC_-carrying isolates carried genes encoding resistance to aminoglycosides, β-lactams, tetracyclines and macrolides. One *E. coli* isolate carried genes conferring resistance to quinolones.

**Table 4:** Antimicrobial resistance gene profile of a selected subset of *bla*_KPC_-positive Enterobacterales.

Species		Antibiotic categories^a^																			
		Amino-glycosides		β-Lactams		Fosfomycin		Phenicols		Rifampicin		Sulfonamides		Tetracyclines		Trimethoprim		Quinolones		Macrolides	
		+	–	+	–	+	–	+	–	+	–	+	–	+	–	+	–	+	–	+	–
*Klebsiella pneumoniae* (*n* = 60)	*n*	57	3	60	0	60	0	5	55	16	44	23	37	15	45	60	0	59	1	6	54
	%	95.0	5.0	100.0	0.0	100.0	0.0	8.3	91.7	26.7	73.3	38.3	61.7	25.0	75.0	100.0	0.0	98.3	1.7	10.0	90.0
*Escherichia coli* (*n* = 9)	*n*	9	0	9	0	6	3	3	6	3	6	7	2	9	0	3	6	1	8	9	0
	%	100.0	0.0	100.0	0.0	66.7	33.3	33.3	66.7	33.3	66.7	77.8	22.2	100.0	0.0	33.3	66.7	11.1	88.9	100.0	0.0

a + indicates that the bacteria carried at least one resistance gene for the antibiotic group; – indicates that the bacteria did not carry any resistance gene for the antibiotic group.

*Klebsiella pneumoniae* harbouring *bla*_KPC_ genes belonged to ST15 and ST11 (Table 5). Notably, while ST15 was predominant, ST11 was observed only in two isolates, both from hospital D (Table 5; Fig. 1).

**Table 5:** Key epidemiological information and sequence types (ST) of *bla*_KPC_ carriers in the four hospitals (A–D)

Strain ID	Species	Hospital	Collection year	Department	Sample type	MLST	Carbapenemase genes			
							KPC	OXA-48	IMP	NDM-1
64	*Klebsiella pneumoniae*	A	2010	ICU	Bronchial fluid	ST15	KPC-2	–	–	–
66	*K. pneumoniae*	A	2010	ICU	Bronchial fluid	ST15	KPC-2	–	–	–
67	*K. pneumoniae*	A	2010	Paediatrics	Bronchial fluid	ST15	KPC-2	–	–	–
84	*K. pneumoniae*	A	2010	Paediatrics	Bronchial fluid	ST15	KPC-2	–	–	–
124	*K. pneumoniae*	A	2010	Paediatrics	Bronchial fluid	ST15	KPC-2	–	–	–
151	*K. pneumoniae*	A	2011	Paediatrics	Bronchial fluid	ST15	KPC-2	–	–	–
152	*K. pneumoniae*	A	2011	ICU	Bronchial fluid	ST15	KPC-2	–	–	–
273	*K. pneumoniae*	A	2011	Paediatrics	Bronchial fluid	ST15	KPC-2	–	–	–
274	*K. pneumoniae*	A	2011	Paediatrics	Bronchial fluid	ST15	KPC-2	–	–	–
278	*K. pneumoniae*	A	2011	Paediatrics	Bronchial fluid	ST15	KPC-2	–	–	–
263	*K. pneumoniae*	A	2011	Paediatrics	Blood	ST15	KPC-2	–	–	–
265	*K. pneumoniae*	A	2011	Paediatrics	Bronchial fluid	ST15	KPC-2	–	–	–
297	*K. pneumoniae*	A	2011	Paediatrics	Bronchial fluid	ST15	KPC-2	–	–	–
294	*K. pneumoniae*	A	2011	Paediatrics	Bronchial fluid	ST15	KPC-2	–	–	–
298	*K. pneumoniae*	A	2011	Paediatrics	Bronchial fluid	ST15	KPC-2	–	–	–
322	*K. pneumoniae*	A	2011	Paediatrics	Bronchial fluid	ST15	KPC-2	–	–	–
326	*K. pneumoniae*	A	2011	Paediatrics	Bronchial fluid	ST15	KPC-2	–	–	–
328	*K. pneumoniae*	A	2011	Paediatrics	Bronchial fluid	ST15	KPC-2	–	–	–
365	*K. pneumoniae*	A	2011	Paediatrics	Bronchial fluid	ST15	KPC-2	–	–	–
342	*K. pneumoniae*	A	2011	Paediatrics	Bronchial fluid	ST15	KPC-2	–	–	–
348	*K. pneumoniae*	A	2011	Paediatrics	Bronchial fluid	ST15	KPC-2	–	–	+
389	*K. pneumoniae*	A	2011	Paediatrics	Bronchial fluid	ST15	KPC-2	–	–	–
408	*K. pneumoniae*	A	2011	Paediatrics	Bronchial fluid	ST15	KPC-2	–	–	–
410	*K. pneumoniae*	A	2011	Paediatrics	Blood	ST15	KPC-2	–	–	–
447	*K. pneumoniae*	A	2011	Paediatrics	Bronchial fluid	ST15	KPC-2	–	–	–
451	*K. pneumoniae*	A	2011	Paediatrics	Blood	ST15	KPC-2	–	–	–
452	*K. pneumoniae*	A	2011	Paediatrics	Blood	ST15	KPC-2	–	–	–
454	*K. pneumoniae*	A	2011	Paediatrics	Bronchial fluid	ST15	KPC-2	–	–	–
455	*K. pneumoniae*	A	2011	Paediatrics	Bronchial fluid	ST15	KPC-2	–	–	–
457	*K. pneumoniae*	A	2011	Paediatrics	Bronchial fluid	ST15	KPC-2	–	–	–
437	*K. pneumoniae*	A	2011	Paediatrics	Bronchial fluid	ST15	KPC-2	–	–	–
529	*K. pneumoniae*	A	2011	Paediatrics	Blood	ST15	KPC-2	–	–	–
580	*K. pneumoniae*	A	2011	Paediatrics	Bronchial fluid	ST15	KPC-2	–	–	–
676	*K. pneumoniae*	A	2012	Paediatrics	Bronchial fluid	ST15	KPC-2	–	–	–
681	*K. pneumoniae*	A	2012	Paediatrics	Blood	ST15	KPC-2	–	–	–
683	*K. pneumoniae*	A	2012	Paediatrics	Blood	ST15	KPC-2	–	–	–
686	*K. pneumoniae*	A	2012	Paediatrics	Bronchial fluid	ST15	KPC-2	–	–	–
724	*K. pneumoniae*	A	2012	Paediatrics	Blood	ST15	KPC-2	–	–	–
725	*K. pneumoniae*	A	2012	Paediatrics	Bronchial fluid	ST15	KPC-2	–	–	–
729	*K. pneumoniae*	A	2012	Paediatrics	Bronchial fluid	ST15	KPC-2	–	–	–
732	*K. pneumoniae*	A	2012	Paediatrics	Bronchial fluid	ST15	KPC-2	–	–	–
847	*K. pneumoniae*	A	2012	Paediatrics	Bronchial fluid	ST15	KPC-2	–	–	–
869	*K. pneumoniae*	A	2012	Paediatrics	Blood	ST15	KPC-2	–	–	–
914	*K. pneumoniae*	A	2012	Paediatrics	Bronchial fluid	ST15	KPC-2	–	–	–
980	*K. pneumoniae*	A	2012	Paediatrics	Bronchial fluid	ST15	KPC-2	–	–	–
1040	*K. pneumoniae*	A	2012	Paediatrics	Bronchial fluid	ST15	KPC-2	–	–	–
1045	*K. pneumoniae*	A	2012	Paediatrics	Bronchial fluid	ST15	KPC-2	–	–	–
1048	*K. pneumoniae*	A	2012	Paediatrics	Blood	ST15	KPC-2	–	–	–
1070	*K. pneumoniae*	A	2012	Paediatrics	Bronchial fluid	ST15	KPC-2	–	–	–
1075	*K. pneumoniae*	A	2013	Paediatrics	Bronchial fluid	ST15	KPC-2	–	–	–
1117	*K. pneumoniae*	A	2013	Paediatrics	Bronchial fluid	ST15	KPC-2	–	–	–
1133	*K. pneumoniae*	A	2013	Paediatrics	Bronchial fluid	ST15	KPC-2	–	–	–
1136	*K. pneumoniae*	A	2013	Paediatrics	Bronchial fluid	ST15	KPC-2	–	–	–
1152	*K. pneumoniae*	A	2013	Paediatrics	Bronchial fluid	ST15	KPC-2	–	–	–
1279	*K. pneumoniae*	A	2013	Paediatrics	Bronchial fluid	ST15	KPC-2	–	–	–
1280	*K. pneumoniae*	A	2013	Paediatrics	Bronchial fluid	ST15	KPC-2	–	–	–
1281	*K. pneumoniae*	A	2013	Paediatrics	Bronchial fluid	ST15	KPC-2	–	–	–
1282	*K. pneumoniae*	A	2013	Paediatrics	Bronchial fluid	ST15	KPC-2	–	–	–
1338	*K. pneumoniae*	A	2013	Paediatrics	Bronchial fluid	ST15	KPC-2	–	–	–
1340	*K. pneumoniae*	A	2013	Paediatrics	Bronchial fluid	ST15	KPC-2	–	–	–
1342	*K. pneumoniae*	A	2013	Paediatrics	Bronchial fluid	ST15	KPC-2	–	–	–
1401	*K. pneumoniae*	A	2013	Paediatrics	Blood	ST15	KPC-2	–	–	+
1407	*K. pneumoniae*	A	2013	Paediatrics	Bronchial fluid	ST15	KPC-2	–	–	+
1403	*K. pneumoniae*	A	2013	ICU	Bronchial fluid	ST15	KPC-2	–	–	–
1406	*K. pneumoniae*	A	2013	ICU	Bronchial fluid	ST15	KPC-2	–	–	–
1408	*K. pneumoniae*	A	2013	Paediatrics	Bronchial fluid	ST15	KPC-2	–	–	–
1503	*K. pneumoniae*	A	2014	Paediatrics	Bronchial fluid	ST15	KPC-2	–	–	–
1508	*K. pneumoniae*	A	2014	Paediatrics	Bronchial fluid	ST15	KPC-2	–	–	–
1512	*K. pneumoniae*	A	2014	Paediatrics	Bronchial fluid	ST15	KPC-2	–	–	–
1577	*K. pneumoniae*	A	2014	Paediatrics	Bronchial fluid	ST15	KPC-2	–	–	–
1587	*K. pneumoniae*	A	2014	Paediatrics	Bronchial fluid	ST15	KPC-2	–	–	–
1591	*K. pneumoniae*	A	2014	Paediatrics	Bronchial fluid	ST15	KPC-2	–	–	–
1592	*K. pneumoniae*	A	2014	Paediatrics	Bronchial fluid	ST15	KPC-2	–	–	–
1663	*K. pneumoniae*	A	2014	Paediatrics	Blood	ST15	KPC-2	–	–	–
1665	*K. pneumoniae*	A	2014	Paediatrics	Blood	ST15	KPC-2	–	–	–
1758	*K. pneumoniae*	A	2014	Paediatrics	Bronchial fluid	ST15	KPC-2	–	–	–
1768	*K. pneumoniae*	A	2014	Paediatrics	Bronchial fluid	ST15	KPC-2	–	–	–
1797	*K. pneumoniae*	A	2014	Paediatrics	Bronchial fluid	ST15	KPC-2	–	–	–
1838	*K. pneumoniae*	A	2014	Paediatrics	Bronchial fluid	ST15	KPC-2	–	–	–
1847	*K. pneumoniae*	A	2014	Paediatrics	Bronchial fluid	ST15	KPC-2	+	–	–
1853	*K. pneumoniae*	A	2014	Paediatrics	Blood	ST15	KPC-2	–	–	–
1859	*K. pneumoniae*	A	2014	Paediatrics	Bronchial fluid	ST15	KPC-2	–	–	–
1893	*K. pneumoniae*	A	2014	Paediatrics	Bronchial fluid	ST15	KPC-2	–	–	–
1902	*K. pneumoniae*	A	2014	Paediatrics	Bronchial fluid	ST15	KPC-2	–	–	–
1909	*K. pneumoniae*	A	2014	Paediatrics	Bronchial fluid	ST15	KPC-2	–	–	+
2030	*K. pneumoniae*	A	2014	Paediatrics	Bronchial fluid	ST15	KPC-2	–	–	–
2054	*K. pneumoniae*	A	2014	Paediatrics	Bronchial fluid	ST15	KPC-2	–	–	–
2057	*K. pneumoniae*	A	2014	Paediatrics	Bronchial fluid	ST15	KPC-2	–	–	–
2059	*K. pneumoniae*	A	2014	Paediatrics	Blood	ST15	KPC-2	–	–	–
2060	*K. pneumoniae*	A	2014	Paediatrics	Bronchial fluid	ST15	KPC-2	–	–	–
13	*K. pneumoniae*	B	2010	ICU	Bronchial fluid	ST15	KPC-2	–	–	–
26	*K. pneumoniae*	B	2010	ICU	Bronchial fluid	ST15	KPC-2	–	–	–
25	*K. pneumoniae*	B	2010	ICU	Sputum	ST15	KPC-2	–	–	–
27	*K. pneumoniae*	B	2010	ICU	Sputum	ST15	KPC-2	–	–	–
32	*K. pneumoniae*	B	2010	ICU	Blood	ST15	KPC-2	–	–	–
33	*K. pneumoniae*	B	2010	ICU	Bronchial fluid	ST15	KPC-2	–	–	+
1203	*K. pneumoniae*	B	2013	Paediatrics	Bronchial fluid	ST15	KPC-2	–	–	–
1202	*K. pneumoniae*	B	2013	Paediatrics	Bronchial fluid	ST15	KPC-2	–	–	–
1800	*K. pneumoniae*	B	2014	ICU	Sputum	ST15	KPC-2	–	–	+
1555	*K. pneumoniae*	C	2014	ICU	Abdominal fluid	ST15	KPC-2	–	–	–
2090	*K. pneumoniae*	C	2015	ICU	Bronchial fluid	ST15	KPC-2	–	–	–
01445	*K. pneumoniae*	D	2014	Cardiology	Urine	ST15	KPC-2	–	–	–
01478	*K. pneumoniae*	D	2014	Neurology	Urine	ST15	KPC-2	–	–	–
01512	*K. pneumoniae*	D	2015	Thoracic Surgery	Blood	ST11	KPC-12	–	–	–
01555	*K. pneumoniae*	D	2015	TB and lung diseases	Sputum	ST15	KPC-2	–	–	–
01557	*K. pneumoniae*	D	2015	TB and lung diseases	Pleural fluid	ST15	KPC-2	–	–	–
01556	*K. pneumoniae*	D	2015	Neurology	Blood	ST11	KPC-14	–	–	–
01567	*K. pneumoniae*	D	2015	TB and lung diseases	Sputum	ST15	KPC-2	–	–	–
01568	*K. pneumoniae*	D	2015	TB and lung diseases	Sputum	ST15	KPC-2	–	–	–
573	*Escherichia coli*	A	2011	Paediatrics	Bronchial fluid	ST709	KPC-2	–	–	+
579	*E. coli*	A	2011	Paediatrics	Bronchial fluid	ST709	KPC-2	–	–	–
769	*E. coli*	A	2012	Paediatrics	Bronchial fluid	ST405	KPC-2	–	–	–
774	*E. coli*	A	2012	Paediatrics	Bronchial fluid	ST405	KPC-2	–	–	+
1402	*E. coli*	A	2013	ICU	Bronchial fluid	ST3580	KPC-2	–	–	–
1593	*E. coli*	A	2014	Paediatrics	Bronchial fluid	ST3580	KPC-2	–	–	–
1942	*E. coli*	A	2014	Paediatrics	Bronchial fluid	ST3580	KPC-2	–	–	–
1793	*E. coli*	A	2014	Paediatrics	Bronchial fluid	ST3580	KPC-2	–	–	–
1808	*E. coli*	A	2014	Paediatrics	Bronchial fluid	ST3580	KPC-2	–	–	–
2060	*E. coli*	A	2014	Paediatrics	Bronchial fluid	ST3580	KPC-2	–	–	–
2052	*E. coli*	A	2014	Paediatrics	Bronchial fluid	ST3580	KPC-2	–	–	–
20	*E. coli*	C	2010	Urology	Urine	ST448	KPC-2	–	–	+
50	*E. coli*	C	2010	Urological Surgery	Urine	ST448	KPC-2	–	–	+

MLST, multilocus sequence typing; ICU, intensive care unit; TB, tuberculosis.

The core genome phylogenetic tree of 60 *bla*_KPC_-carrying *K. pneumoniae* isolates presented only three genotypic groups: two groups had ST15 and one group had ST11 (Fig. 1). The largest ST15 lineage contained all isolates from three hospitals (A, B and C) collected during 2010 and 2015. Interestingly, two resistance gene patterns were observed in this lineage originated from isolates from the three hospitals through the years. In contrast, strains collected from hospital D constituted two separate ST15 and ST11 groups, sharing a quite similar resistance gene profile.

KPC-producing *E. coli* had more diverse sequence types, including ST3580, ST448, ST709 and ST405 (Table 5), corresponding to four genotypic groups in its core genome phylogenetic tree (Fig. 2). The resistance profile was similar among *E. coli* strains belonging to the same sequence type.

### 3.3 Characterisation of bla_KPC_-carrying plasmids and genetic environment of bla_KPC_

**Fig. 3:**
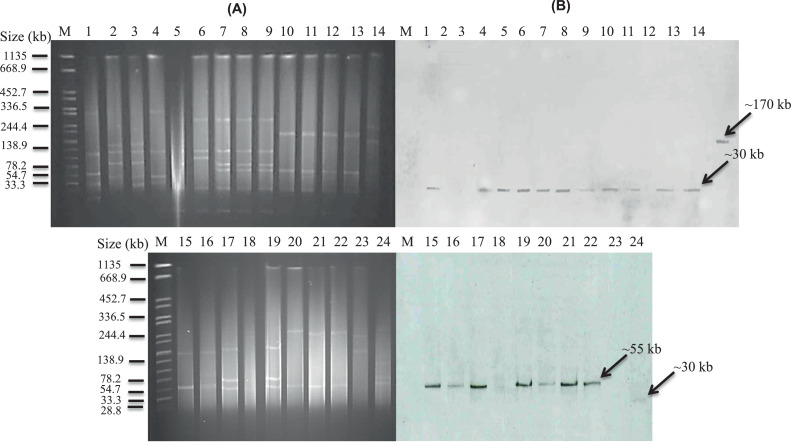
*S1* nuclease pulsed-field gel electrophoresis (S1-PFGE) and Southern blotting of plasmids carrying *bla*_KPC_ from clinical isolates. (A) DNA fingerprint of *S1*-treated plasmid DNA of selected Enterobacterales from clinical isolates stained with ethidium bromide. (B) Autoradiogram of gel A showing plasmids carrying the *bla*_KPC_ gene. M, *Salmonella* Braenderup H9812 (molecular weight marker); lanes 1 and 4–8*, Klebsiella pneumoniae* isolates from hospital A; lanes 2 and 3*, Escherichia coli* isolates from hospital A; lanes 9–13, 23 and 24, *K. pneumoniae* isolates from hospital B; lane 14, *K. pneumoniae* from hospital C; lanes 15–19, *K. pneumoniae* isolates from hospital D; lanes 20 and 21, *K. pneumoniae* from hospital C; lane 22*, E. coli* isolates from hospital C.

Randomly selected *bla*_KPC_-positive isolates (*n* = 24) from the four hospitals were subsequently analysed by S1-PFGE and Southern blotting for *bla*_KPC_, showing that most (21/24) *bla*_KPC_ genes were plasmid-borne (Fig. 3). Interestingly, the size of *bla*_KPC_-carrying plasmids in hospital A and B was similar (∼30 kb). In contrast, *bla*_KPC_-positive *K. pneumoniae* in hospitals C and D carried plasmids different in size: ∼170 kb and ∼55 kb, respectively. Two *bla*_KPC_-positive *E. coli* isolates and one *K. pneumoniae* isolate did not hybridise. IncFIB(K), IncN and IncFIIK were predominant plasmid types among *bla*_KPC_-carrying plasmids.

**Fig. 4:**
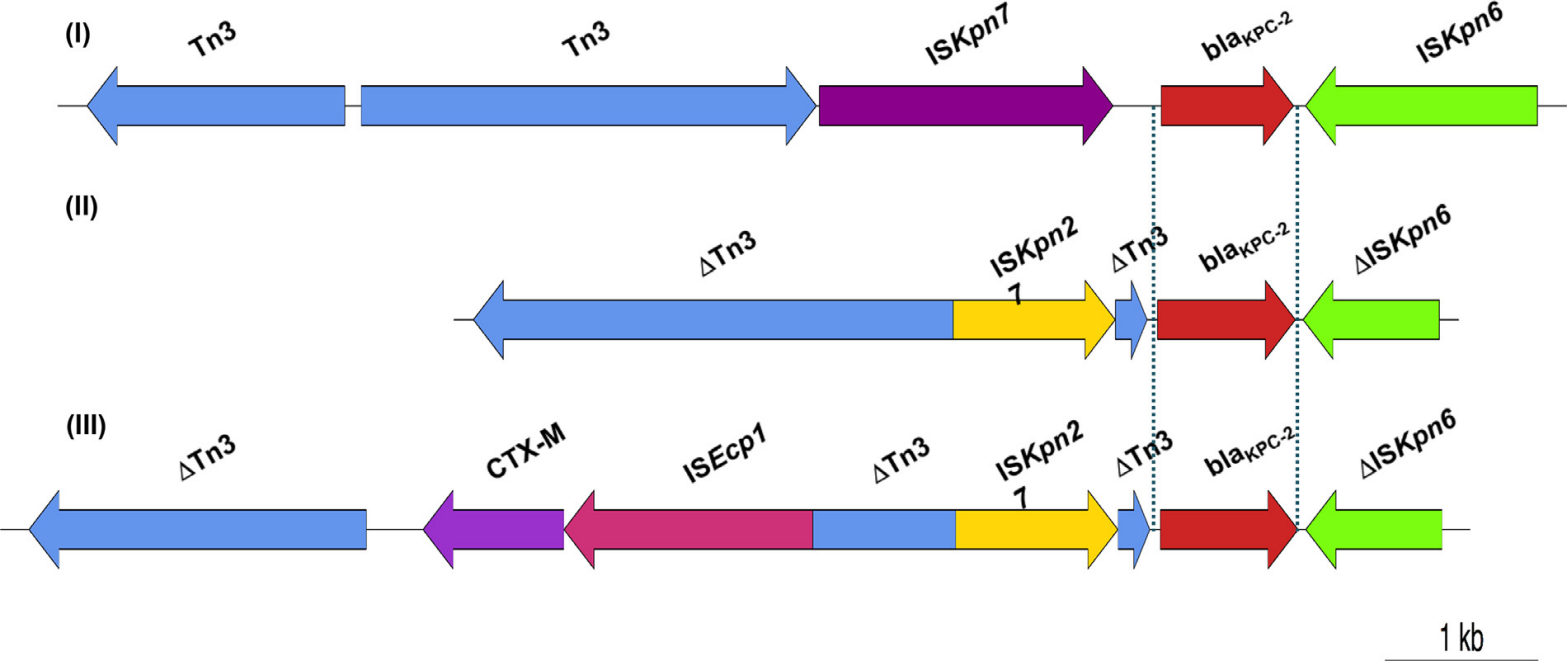
Gene context models of *bla*_KPC-2_ in *Escherichia coli* and *Klebsiella pneumoniae* isolates*.* (I) Model observed in *K. pneumoniae* and *E. coli* isolates carrying 55-bp or 30-bp plasmids. (II) Model observed in *K. pneumoniae* isolates carrying 55-bp or 170-bp plasmids. (III) Model observed in *K. pneumoniae* isolates carrying 55-bp plasmids.

Analysis of the genetic environment of the *bla*_KPC-2_ gene revealed various genetic contexts of *bla*_KPC-2_ (Fig. 4) in different plasmids. Tn*4401b* isoform harbouring the *bla*_KPC-2_ gene was present in several *E. coli* and *K. pneumoniae* isolates carrying 55-bp or 30-bp plasmids with no deletion between IS*Kpn7* and *bla*_KPC-2_ (Model I). Two different structures of the *bla*_KPC-2_ gene environment were observed among *K. pneumoniae* isolates. The first variant possessed IS*Kpn27* upstream and a truncated IS*Kpn6* downstream of the *bla*_KPC-2_ gene (Model II), whereas the other had another mobile element containing IS*Ecp1*–*bla*_CTX-M_ inserted into a Tn*3* transposon at 950 bp upstream of IS*Kpn27* (Model III).

## 4 Discussion

Since their first detection in the USA [17], *bla*_KPC_-carrying plasmids have rapidly spread across countries and continents and the *bla*_KPC-2_ gene was recently described in several hospitals in Vietnam [4,[6], [7], [8], [9]]. Here we report the widespread distribution of *bla*_KPC_-producing Enterobacterales from samples collected from patients admitted to four large hospitals between 2010 and 2015 in Hanoi, Vietnam, with the first isolate detected in early 2010. It is possible there was already undetected circulation prior to 2010. *bla*_KPC_ genes were detected in 122 (20.4%) of 599 CRE isolates. Co-expression of *bla*_KPC_ and multiple other β-lactamase-encoding genes was found here as reported previously [6], [7], [8].

The most dominant sample type was bronchial fluid usually collected from mechanically ventilated patients, which are commonly found to be associated with nosocomial infections [18]. Genes conferring resistance to different antibiotic categories were also observed in other CRE isolated in Vietnam [5,8]. This shows the increasing number of diverse resistant strains in hospital settings in Vietnam, which pose a great challenge for doctors in efficient antibiotic selection.

The predominance of ST15 in the four hospitals was similar to other published data in Vietnam and Asian countries including China, which shares a >1000 km border and trading and tourism activities with Vietnam [6,8,19,20]. ST15 *K. pneumoniae* has been reported worldwide as a clone carrying multiple carbapenemase genes, which was also observed in other studies in Vietnam [8,21]. Carbapenemase-producing ST15 *K. pneumoniae* was first reported in Vietnam in samples collected between 2014–2015 [6]; however, in our study they were found in samples collected since 2010, facilitating the hypothesis of their presence in Vietnam hospitals prior to 2010. All *bla*_KPC_-carrying *K. pneumoniae* isolated from hospital A throughout the study period belonged to ST15, suggesting that they were resident flora. Although ST11 was reported among carbapenem-resistant *K. pneumoniae* in Vietnam previously [22,23], it was detected in only two isolates in the current study. This sequence type has also been associated with nosocomial outbreaks in several countries, especially in China [21,24,25]. Isolates from hospitals A, B and C belonged to the same lineage, supporting the hypothesis of exchange of bacteria and plasmids between these hospitals via patient transfer. Hospital D served a different targeted patient population and did not transfer patients with the other hospitals, which might explain the finding that resistant strains collected here evolved in their own ways forming two separate lineages.

Plasmid types carrying *bla*_KPC_ genes found in this study were diverse and similar to those in many countries such as the UK, USA and China [26], [27], [28]. Four plasmid sizes were detected in this study that differed from the 150-kb *bla*_KPC_-carrying plasmid observed previously in Vietnam [6]. Notably, *bla*_KPC_-carrying plasmids isolated from hospital D all had the same size (∼55 kb) but belonged to two different ST groups (ST11 and ST15). This evidence supports the independent existence of *bla*_KPC_-carrying plasmids and, together with the diversity of sizes of *bla*_KPC_-carrying plasmids, shows the possibility of plasmid transmission across bacterial strains and species.

Regarding the gene context models of *bla*_KPC-2_, the Tn*4401* isoform is endemic in many countries, whereas it is infrequently observed in China. Instead, IS*Kpn27*–*bla*_KPC_–ΔIS*Kpn6* within the Tn*3* transposon frame accounted for the majority of isolates from China but not in other countries, which was also found in our study (Model II) [21,29]. This model was not only observed in Enterobacterales such as *E. coli, K. pneumoniae* and *Citrobacter freundii* but was also detected in *P. aeruginosa* [29], [30], [31], showing the possibility of plasmid transmission between different species and genera of bacteria. All isolates carrying this model in our study showed high resistance to carbapenems (MIC ≥ 8 μg/mL), except for one isolate maintaining susceptibility to imipenem.

Model III with the combination of IS*Ecp1*–*bla*_CTX-M_ and IS*Kpn27*–*bla*_KPC_–ΔIS*Kpn6* in the same plasmid (Fig. 4) has not been reported before. However, the co-existence of one plasmid carrying IS*Ecp1*–*bla*_CTX-M_ and one plasmid carrying IS*Kpn27*–*bla*_KPC_–ΔIS*Kpn6* in one isolate was reported from China in 2010 [32]. This suggests that a recombination event occurred bringing these two structures into one plasmid during the evolution of the isolate/mobile gene element. Interestingly, MIC results showed that this combination confers only weak resistance to carbapenems but still strong resistance to cefotaxime. Insertion of IS*Ecp1*–*bla*_CTX-M_ into the IS*Kpn27*–*bla*_KPC_–ΔIS*Kpn6* frame might affect the phenotype of carbapenem resistance.

This study has several limitations. We do not have full hospital denominators and only a limited amount of metadata were collected, and we do not know patient treatment outcomes. Therefore, we were unable to draw further conclusions on the epidemiological characteristics (such as burden of disease, distinction of community-acquired and hospital-acquired isolates, patient-to-patient and environmental persistence, commensal and pathogenic bacteria) of *bla*_KPC_-expressing Enterobacterales in these hospitals.

In conclusion, we describe the widespread presence of *bla*_KPC_-expressing Enterobacterales in four large hospitals in Hanoi, Vietnam, since 2010, which may have started earlier, along with their resistance patterns, sequence types, genotypic relationship, plasmid sizes and genetic context. The spread of these carbapenemase-producers adds an additional challenge to the treatment of diseases caused by these common bacteria with a very extensive expression of genes conferring additional resistances.

Our study also provides evidence for the likelihood of KPC-producer circulation among three of the four hospitals as well as the possibility of plasmid transmission across bacterial strains and species. Data from this study contribute to a more comprehensive picture of the antimicrobial resistance situation in hospitals in Hanoi in the context of overcrowding and lack of hospital infection control programmes.

## Funding

This work was supported by a grant from the Newton Fund Vietnam [MOST: NHQT/SPDP/02.16], a Grant-in-Aid for the Research Program on Emerging and Re-emerging Infectious Diseases [JP20fk0108061 for KS; JP20fk0108093, JP20fk0108139 and JP20wm0225008 for MS] from the Japan Agency for Medical Research and Development (AMED), the Wellcome Trust of Great Britain, UK, and the IRD and LMI DRISA.

## Ethical approval

The samples used in this study were taken from the Isolate Bank of the National Institute of Hygiene and Epidemiology (NIHE). This study is part of the main project ‘Assessing the impact and burden of antimicrobial resistance in Vietnam, genomic characterization and risk factors related to antimicrobial resistance of common bacteria in Vietnam’, which was approved by the institutional review board (IRB) of NIHE [IRB code IRB-VN01057-38/2016]. Individual informed consent was waived because of the retrospective nature of the work and because no personal identifiers were collected.

## Declaration of Competing Interest

None declared.
